# A non-surgical approach to the management of lumbar spinal stenosis: A prospective observational cohort study

**DOI:** 10.1186/1471-2474-7-16

**Published:** 2006-02-23

**Authors:** Donald R Murphy, Eric L Hurwitz, Amy A Gregory, Ronald Clary

**Affiliations:** 1 Rhode Island Spine Center, 600 Pawtucket Avenue, Pawtucket, RI 02860, USA; 2 Department of Community Health, Brown University School of Medicine, USA; 3 Research Department, New York Chiropractic College, USA; 4 Department of Public Health Sciences and Epidemiology, John A. Burns School of Medicine University of Hawaii, Manoa, Hawaii, USA

## Abstract

**Background:**

While it is widely held that non-surgical management should be the first line of approach in patients with lumbar spinal stenosis (LSS), little is known about the efficacy of non-surgical treatments for this condition. Data are needed to determine the most efficacious and safe non-surgical treatment options for patients with LSS. The purpose of this paper is to describe the clinical outcomes of a novel approach to patients with LSS that focuses on distraction manipulation (DM) and neural mobilization (NM).

**Methods:**

This is a prospective consecutive case series with long term follow up (FU) of fifty-seven consecutive patients who were diagnosed with LSS. Two were excluded because of absence of baseline data or failure to remain in treatment to FU. Disability was measured using the Roland Morris Disability Questionnaire (RM) and pain intensity was measured using the Three Level Numerical Rating Scale (NRS). Patients were also asked to rate their perceived percentage improvement.

**Results:**

The mean patient-rated percentage improvement from baseline to the end to treatment was 65.1%. The mean improvement in disability from baseline to the end of treatment was 5.1 points. This was considered to be clinically meaningful. Clinically meaningful improvement in disability from baseline to the end of treatment was seen in 66.7% of patients. The mean improvement in "on average" pain intensity was 1.6 points. This did not reach the threshold for clinical meaningfulness. The mean improvement in "at worst" pain was 3.1 points. This was considered to be clinically meaningful.

The mean duration of FU was 16.5 months. The mean patient-rated percentage improvement from baseline to long term FU was 75.6%. The mean improvement in disability was 5.2 points. This was considered to be clinically meaningful. Clinically meaningful improvement in disability was seen in 73.2% of patients. The mean improvement in "on average" pain intensity from baseline to long term FU was 3.0 points. This was considered to be clinically meaningful. The mean improvement in "at worst" pain was 4.2 points. This was considered to be clinically meaningful. Only two patients went on to require surgery.

No major complications to treatment were noted.

**Conclusion:**

A treatment approach focusing on DM and NM may be useful in bringing about clinically meaningful improvement in disability in patients with LSS.

## Background

Lumbar spinal stenosis (LSS) is a common and often disabling disorder that generally occurs in the sixth or seventh decade of life [1], although it can uncommonly occur in younger individuals [2]. The incidence of this condition has been reported to be 8–11% [3], with a slight preponderance in women [1]. LSS can lead to low back and leg pain, most typically via encroachment on the central canal, lateral recess, or lateral canal. The source of the encroachment is typically vertebral body osteophytes, hypertrophy of the ligamentum flavum or zygapophyseal joint, or a combination of these [1]. The posterior longitudinal ligament may be involved in some individuals [4]. The development of these degenerative changes is often accompanied by restriction of segmental mobility [1].

One of the hallmarks of LSS is neurogenic claudication, in which the patient develops low back and/or leg pain after a period of walking that progressively worsens as walking is continued, with improvement or resolution when walking ceases and the patient sits or flexes the lumbar spine [5].

LSS is one of the most common reasons for spine surgery in older people [6], although little is known about the efficacy of surgical management of patients with LSS, particularly compared to non-surgical management [7]. It is generally felt that most patients with LSS should be managed non-surgically before considering surgical intervention [8], but little is also known about what non-surgical approaches are most efficacious.

LSS can involve the central canal, the lateral recess, the lateral canal, or any combination of these [6]. This can lead to nerve root pain and dysfunction, i.e., radiculopathy. The pathophysiology of radiculopathy secondary to LSS is different from that of radiculopathy secondary to herniated disc (HD). In recent years it has increasingly become clear that much of the pain with acute radiculopathy secondary to HD is chemical, not compressive in nature [9,10]. The chemical inflammatory process with HD is initiated by the presence of nuclear material. But with LSS, it is likely that a different, or additional, mechanism that is involved in the production of nerve root pain.

Experimental evidence has suggested that chronic compression of the nerve root in LSS causes compromise of blood flow leading to congestion, ischemia, and intraneural edema [11]. This then leads to the development of periradicular fibrosis [12]. Increased pain with walking that is relieved with lumbar flexion (neurogenic claudication) is one of the hallmarks for LSS. Neurogenic claudication likely arises from increased metabolic demands of the nerve root in the presence of vascular compromise [13] and traction on the adhesed nerve root when lower extremity movement occurs during walking [14]. This may explain why the SLR is often negative in pts with LSS [8], but is typically positive in patients with herniated disc. With LSS, compression, vascular compromise and perineural fibrosis dominate the pathophysiological picture, thus maneuvers that increase IVF pressure, i.e., extension [15], or increase metabolic demands of the nerve root and movement of the fibrotic nerve root, as with walking, exacerbate the pain.

A non-surgical approach that attempts to target the unique pathophysiology of LSS may be best able to rapidly improve pain and function in these patients. Such a treatment strategy would attempt to mobilize the segment(s) involved, decompress the involved nerve root(s) and mobilize the involved nerve root(s) to break up periradicular adhesion, thus releasing nerve root entrapment, and restoring vascular function. It would appear that maintaining intersegmental and nerve root mobility would then be important in order to maximize the long term benefit of treatment.

The purpose of this study was to assess, using rigorous outcome measures, the results of a non-surgical management strategy for patients with LSS that focuses on distraction manipulation (DM) and neural mobilization (NM). Theoretically, these methods were employed in order to improve motion segment mobility (DM) and nerve root mobility (NM). It is not known whether these modalities actually create these effects, and this study does not evaluate these theoretical mechanisms. But the outcome of a strategy that focused on these methods was assessed. This strategy has not previously been evaluated.

## Methods

Data were gathered on all consecutive patients meeting the inclusion criteria who were seen from 5/17/00 to 9/19/03. Inclusion criteria were:

1. Leg Pain with or without low back pain

2. MRI or CT documented lateral canal, lateral recess or central canal stenosis, alone or in combination, in the lumbar spine or clear neurogenic claudication as determined by history, i.e., a description by the patient of the onset of leg pain with walking that steadily increased with continued walking, and resolved or improved with rest with the spine in flexion.

3. All patients must remain in treatment for at least one re-exam (typically performed every 3–4 weeks) and complete the prescribed course of treatment.

4. Ability to speak English

Exclusion criteria were:

1. Radiculopathy by other causes such as herniated disc

2. Claudication symptoms determined to be vascular

3. Systemic illness

4. Contraindications to the study treatments

5. Unable to communicate well in English

6. Worker's Compensation/Personal Injury cases

### Outcome Measures

• Disability was measured using the Roland Morris Low Back Pain and Disability Questionnaire (RM) [16].

• Pain intensity was measured using the Three Level Numerical Rating Scale (NRS) [17]. With this, each patient was given an form on which three scales were supplied ranging from 0 (no pain) to 10 (unbearable pain). These scales allowed the patient rated pain intensity at the moment, on average, and at its worst.

• Patients were asked to rate their perceived percentage improvement. That is, patients were provided with a numerical scale ranging from 0% (no improvement) to 100% (complete resolution) and were asked to circle the numerical value that best represented their level of improvement, if any.

Outcome measurements were performed at baseline and again at each scheduled re-examination visit, usually every 3–4 weeks until the end of treatment. As this was a pragmatic study that assessed the outcome of patients treated with the usual protocol utilized at the Rhode Island Spine Center, no set number of treatments, or set duration of treatment, was assigned. Rather, patients were treated in the manner in which the treating chiropractors in the study (DRM and AAG) would normally utilize in every day practice.

FU measures were then obtained by a research assistant by phone or mail for long term FU.

Other data gathered included age, sex, date of last visit, duration of symptoms, primary diagnosis, secondary diagnosis (if any), rheumatologic or orthopedic conditions affecting the spine, number of visits, levels affected based on MRI or CT, history of lumbar surgery and type, treatments applied in the clinic, concomitant treatments outside the clinic, and complications to the study treatments.

### Interventions

Patients were treated according to the usual protocol utilized at the Rhode Island Spine Center for patients with radiculopathy secondary to LSS. The primary interventions, which were utilized in all patients, were:

- Distraction manipulation (DM) – This is a manipulative technique developed by Cox [18]. Although other forms of manipulation are believed to be effective in patients with LSS [19], no form other than DM was used with the patients in this study.

**Figure 1:**
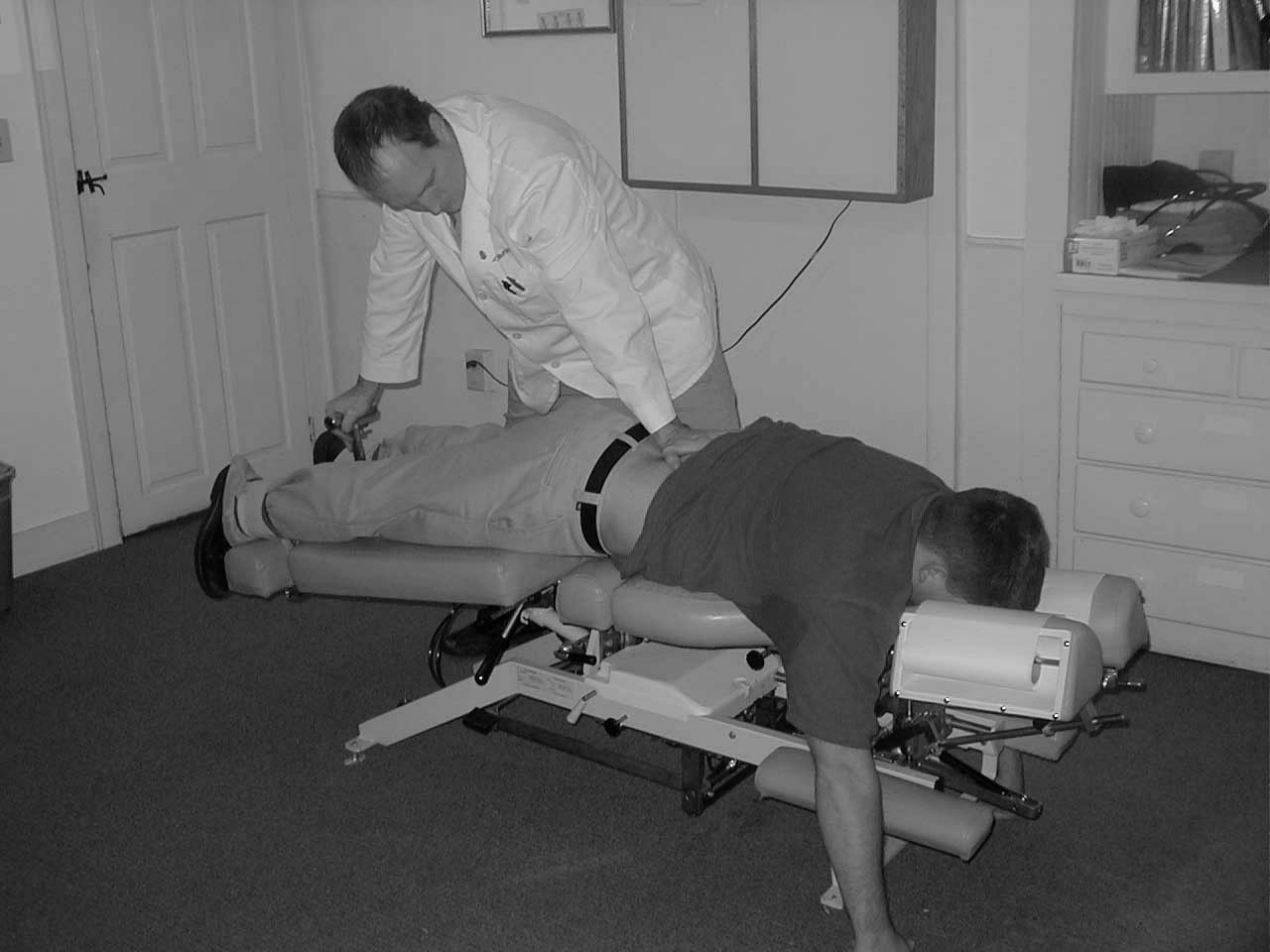
The application of distraction manipulation.

In applying DM, the patient lay prone on a table that allows for distraction of the spine through inferiorward and flexion movement of the lower body (figure 1). This maneuver has been demonstrated to decrease intradiscal pressure [20] and is believed to create vertebral motions and increase the intervertebral foramen [21].

- Neural mobilization (NM) – This a manual and exercise oriented method that is theorized to mobilize nerve roots that are suspected to be the source of nerve root pain [22,23]. Distal mobilization was applied by having the patient lie supine while the doctor or therapist dorsiflexed the ankle and flexed the hip with the knee extended. The leg was raised until the practitioner felt the "barrier" [24], i.e., the point at which tension is initially felt. The foot is then moved alternately into plantar flexion and dorsiflexion repeatedly for several cycles.

- Exercises that are taught to the patient and which are designed to compliment the DM and NM by mobilizing the lumbar spine and the involved nerve root(s). These included the "cat and camel" exercise [25] in which the patient is quadruped and alternately flexes and extends (within the comfort level) the cervical and lumbar spine, and "nerve flossing" exercises [25], which attempt to mobilize the involved nerve roots and there associated peripheral nerves.

DM and NM and the related exercises are the constants of treatment in this study – all patients are treated with these methods. In addition, certain patients may also have been had other modalities included in their individual programs, such as mobilization exercises and spinal stabilization exercise [26,27]. While the frequency and duration of care were determined on an individual basis, patients were generally seen 2–3 times per week for 3 weeks initially, after which the first follow up (FU) reexamination was performed, which included the primary outcome measures (see below). This was typically followed by either continued frequency of 2 times per week or a reduction in frequency to 1 time per week, though some patients who are fully recovered were released after the first FU reexamination to 3 week FU.

This was a practice-based project in which the data gathered were those data that are collected as part of the routine of practice at the Rhode Island Spine Center. Also, the treatments provided each patient were those that are provided in the routine care of patients with LSS at the Rhode Island Spine Center. No experimental procedures were used and no personally identifiable information on any patient is presented. The study protocol was reviewed and approved by the HIPAA compliance officer of the Rhode Island Spine Center. Because of this, it was not deemed necessary to obtain formal approval from an Institutional Review Board.

### Statistical analysis

Descriptive statistics, including frequencies and proportions for categorical variables, and means, standard deviations, and ranges for continuous variables, were computed. Improvements in current, average, and worst pain (NRS) and disability (RM) from baseline to the last follow-up re-examination and to the long-term assessment were measured by computing change scores for each case and taking the group mean, as well as by computing percent change in each outcome variable ([baseline score – follow-up score]/baseline score). 95% confidence intervals around the change scores were computed; paired t-tests were used to assess the statistical significance of the change scores. A three point or greater improvement on the RM was deemed clinically meaningful [28-30]. Data were stratified by sex in an a priori subgroup analysis.

## Results

Table 1 presents the sociodemographic and baseline clinical characteristics of the patients.

Data were gathered prospectively on 57 consecutive patients, 36 female and 19 male. Two patients were excluded for lack of baseline data or failure to remain in treatment until the first FU reexamination. The mean age was 65.2 years. The mean duration of symptoms prior to starting treatment was 134.2 weeks. Three patients had a history of previous lumbar spine surgery. For the 43 patients in whom imaging was available, lateral canal or lateral recess stenosis was present in 33 patients and central canal stenosis was present in 28 patients. The majority of patients had both lateral and central stenosis. The most common level of involvement was L4-5 (34 patients). The next most common level of involvement was L3-4 (20 patients) followed by L5-S1 (16 patients), L2-3 (seven patients) and L1-2 (one patient). One patient had involvement at all lumbar levels. The majority of patients had involvement at more than one level. In the remaining patients, LSS was established by the presence of low back pain and leg pain in an older individual with a clear history of neurogenic claudication.

All patients were treated with DM, as close to the at the level(s) of LSS as possible, and neural mobilization, attempting to target the nerve root(s) involved. Twenty-nine patients were taking some form of oral medication (non-steroidal anti-inflammatory drugs, muscle relaxants, analgesics) at intake. No attempt was made to alter medication usage and continued medication usage after intake was not recorded, thus it is impossible to know how long each patient continued on his or her medication. Two patients were referred for epidural steroid injections.

The mean total number of treatments was 13.3 (range 2–50). This included visits to both the chiropractic physician and physical therapist. Forty-four patients were reached for long term FU. The mean duration of FU was 16.5 months (range 3–48 months).

The main results are presented in Table 2.

Statistically significant and clinically meaningful changes were observed in the mean patient-rated percentage improvements from baseline to the end to treatment (95% CI 55.9–74.2; P < 0.0001) and from baseline to long term FU (95% CI 21.1–61.9; P = 0.0002). The mean improvement in disability as measured by the RM score from baseline to the end of treatment was 5.1 points (95% CI 3.4–6.8; P < 0.0001) and from baseline to long term FU was 5.2 points (95% CI 3.0–7.3; P < 0.0001), each exceeding the three points that has been estimated to be the threshold for clinically meaningful improvement using the RM [28-30]. The mean percentage improvements in disability from baseline to end of treatment (95% CI 24.5–59.6; P < 0.0001) and from baseline to long term FU (95% CI 21.1–61.9; P = 0.0002) were also statistically and clinically significant. Of the 48 patients in whom data were available regarding improvement in disability as measured by the RM, clinically meaningful improvement (i.e., 3+ points [28-30]) was seen in 32 patients (66.7%). For long term FU, among the 41 patients for whom these data were available, clinically meaningful improvement in disability was observed in 30 (73.2%).

Mean and mean percentage improvements in pain intensity "currently", "on average" and "at its worst" from baseline to end of treatment and from baseline to long term FU were all statistically significant. All were also clinically meaningful (i.e. 2 points or greater[31]) with the exception of pain intensity "currently" from baseline to the end of treatment and "on average" from baseline to the end of treatment (table 2). Only two patients went on to require surgery.

No major complications to treatment were seen in any patient. Transient increased pain was seen in 12 patients, nine after treatment with DM and NM (in one, this occurred on two occasions), two after home exercise for NM and one in which a patient had increased leg pain after falling asleep on an ice pack. In all cases, the increased pain was minor and transient.

## Discussion

The results of this study suggest that the combination of DM and NM may be useful for patients with LSS. Interpretation of the results must be made with caution, however, as the absence of randomization and control does not eliminate the possibility of treatment bias and does not allow one to distinguish these outcomes from those that would result from natural history. However, in a natural history study by Johnsson [32], 70% of patients were found to be unchanged over 49 months, and 15% improved. So it would appear from this that, while deterioration is not the norm in patients with SS, most patients would not be expected to improve over time.

Nonetheless, clinically meaningful improvement in disability was seen in over two-thirds of the patients, and the improvement appeared to be maintained over an average of 16.5 months after cessation of treatment. The sustained improvement over the long term may relate to the emphasis in the management strategy on treatments that are designed to specifically address the known pathophysiology of LSS and on exercise that was designed to compliment the DM and NM, with continuous monitoring of compliance with home exercise throughout the treatment process. However, this study's design does not allow for firm conclusions to be drawn regarding this. Only two patients went on to require surgery, suggesting that the treatment approach studied here may be an effective alternative to surgery for patients with LSS.

This study can be examined in the light of other studies that have looked at the effectiveness of non-surgical management of LSS, some of which have compared it to surgery. In the Maine Lumbar Spine Study [33], Atlas, et al compared non-surgical management, which was not strictly defined, with surgery in 141 patients with LSS. They found that at one year FU, the non-surgically treated group had only improved by an average of 1.6 points on the RM, compared to the surgical group, which improved by an average of 8.4 points. At four years, the non-surgical group improved an average of 3.5 points on the RM, while the surgical group had improved by an average of 8.5 points [34]. This is contrasted with the 5.2 point improvement seen in the present study as a result of the non-surgical approach taken here. Simotas, et al [35] followed 49 patients treated with a non-surgical approach that included oral non-steroidal or steroidal medication and epidural steroid injection along with postural instruction and mobilization and stabilization exercises. While RM data was collected at baseline, no FU data on the RM are provided. As such, it is difficult to directly compared the Simotas, et al study with the present one. However, they found that nine out of 49 patients (18.5%) required surgery, compared to two out of 44 patients (4.5%) in the present study.

From this, it is reasonable to conclude that the treatment approach in this study is a viable alternative to surgery for patients with LSS, and compares favorably with other non-surgical approaches that have been studied. As the efficacy of surgery does not appear to decrease if it is delayed in favor of a non-surgical trial [36], most patients with LSS should be treated non-surgically for a period of time before considering operation. DM and NM may be one non-surgical option that can be offered to patients.

The management strategy in this study focused on two treatment modalities, DM and NM. DM may have benefits that relate to biomechanical effects on the stenotic segment, such as reduction of intradiscal pressure [20] or widening of the space in the region of the nerve root [21]. DM may also have neurophysiological effects that may be helpful in patients with LSS, such as facilitation of afferent input from mechanoreceptors [37], possibly helping to improve proprioception, which has been shown to be impaired in patients with LSS [38], or hypoalgesia [39]. While most of the studies on the neurophysiological effects of spinal manipulation do not specifically assess DM, evidence suggests that DM has similar neurophysiological effects as other forms of manipulation [40].

NM is hypothesized to gently move both the anatomical structures proximate to the neural elements that are being compromised, as well as the neural elements themselves [22]. This may help patients with LSS by releasing perineural adhesions, thus decreasing traction strain on the nerve root, especially with walking. A great deal more basic science research into the effects of both DM and NM is needed to investigate these proposed mechanisms.

No major complications were seen in any patient, and transient, mild increase in symptoms was seen in 12 patients (21.8%). This is actually less than the 34–55% rate of transient pain related to manipulative treatment in general that has been reported in the literature [41,42]. However, rare complications may not be detected in a sample size such as this one, so larger samples will be required to further investigate the safety of this approach to patients with LSS.

These patients were treated an average of 13.3 times. This study does not allow one to draw conclusions about the optimum number of treatments for patients with LSS. However, it can reasonably be said that the 13.3 treatments likely reflects an adequate number of treatments. This may help the treating clinician in decision making regarding how long to continue to treat a patient with LSS using this approach. It would appear that a decrease of three points on the RM instrument is a good indicator of clinically meaningful improvement in patients with LSS [28-30]. Thus, if, after 13 or so treatments, a minimum improvement of three points on the RM questionnaire is not seen, further treatment with DM and NM is not warranted. It must be noted that a relatively wide range of treatment visits (2–50) was seen in this study, suggesting that individual differences in patient responses to treatment exists which necessitates greater or fewer than the mean number of treatments Nonetheless, 49 of 54 patients (91%) required between seven and 18 visits. So, from the data presented here, it can be concluded that the number of treatments required should be within six visits of the mean in the majority of patients.

This study is useful in that it assesses the outcome of a treatment approach in a "real world" environment. That is, the patients in this practice-based study were treated as they would be under normal circumstances according to the protocol for patients with LSS utilized at the Rhode Island Spine Center. There was no attempt to control the number of visits or the types of treatments that were provided in addition to DM and NM. As such, however, there is no way to tell the extent to which any particular treatment contributed to the outcome in each case. Also, interpretation of the results with regard to efficacy is not possible because of the absence of randomization and appropriate control group(s). The data presented here do suggest, however that the combination of DM and NM may be a useful approach for patients with LSS, and that further investigation in the form of randomized, controlled trials is warranted.

Limitations of this study include, as was stated earlier, absence of randomization and appropriate control groups. But this study was designed as an observational study to undertake preliminary investigation of the use of DM and NM in the management of patients with LSS. Proper randomized, controlled trials are feasible in this area. Also, twelve patients from this series did not have imaging confirmation of the presence of LSS and the diagnosis was made based on the presence of low back pain and/or leg pain in an older person with a clear history consistent with neurogenic claudication. It is felt that including these patients in the study is appropriate in that neurogenic claudication is so characteristic of LSS [5], that it is very unlikely that any other condition would be causing the pain in these patients. Finally, the average pain intensity from baseline to the end of treatment was only 1.6 points. A change in 2 points on the NRS is generally considered to be the threshold for clinically meaningful improvement [31]. However, in spite of this, a clinically meaningful change in disability was seen from baseline to the end of treatment. Also, there was a mean 3 point change in average pain intensity from baseline to final FU. This change can be considered to be clinically meaningful.

## Conclusion

The combination of DM and NM may be a safe and effective approach for patients with LSS. Because the sample size is relatively small and there is no control group, firm conclusions regarding this cannot be drawn. The outcome of this approach compares favorably with other non-surgical treatments, and treatment with DM and NM may be a viable non-surgical option before considering surgery for LSS. This approach deserves closer scrutiny in the form of randomized controlled trials.

## Compteting interests

The author(s) declare that they have no competing interests.

## Authors' contributions

DRM conceived of the idea of compiling the data for publication, was one of the treating practitioners and was the principle author of the manuscript.

ELH was responsible for statistical analysis, helped with design and presentation, and contributed to the writing of the manuscript.

AAG was one of the treating practitioners, helped with data gathering and organization, and contributed to the writing of the manuscript.

RC helped with data gathering and organization and contributed to the writing of the manuscript.

## Pre-publication history

The pre-publication history for this paper can be accessed here:



## Figures and Tables

**Table 1:** Baseline characteristics.

Variable	N	Mean	Median	Standard Deviation	Range
Age	55	65.2	67.0	9.6	32.0–80.0
Male	19	-	-	-	-
Female	36	-	-	-	-
Duration (weeks)	55	134.2	26.0	299.3	1.0–1560
RM score baseline	51	10.6	11.0	4.5	2.0–20.0
Current Pain baseline	54	3.9	3.3	2.9	0–9.0
Average Pain baseline	53	5.2	5.0	2.6	0–10.0
Worst Pain baseline	54	8.7	9.0	2.0	5.0–10.0

Legend: RM – Roland Morris Low Back Pain and Disability questionnaire; FU – follow up

**Table 2:** Outcomes from baseline to last re-exam and to long-term follow-up.

Variable	Last FU					Long Term FU				
	N	Mean	Median	95% CI	P value	N	Mean	Median	95% CI	P value

Change in RM Sore	48	5.1	4.0	3.4 – 6.8	<0.0001	41	5.2	5.0	3.0 – 7.3	<0.0001
Change in Current Pain	49	1.8	2.0	0.9 – 2.7	0.0002	45	2.1	2.0	1.0 – 3.1	0.0003
Change in Average Pain	47	1.6	1.0	0.6 – 2.7	0.0024	44	3.0	3.0	2.0 – 4.1	<0.0001
Change in Worst Pain	47	3.1	2.0	2.0 – 4.3	<0.0001	45	4.2	6.0	2.8 – 5.5	<0.0001
Self Rated Improvement	52	65.1	80.0	55.9 – 74.2	<0.0001	44	75.6	90.0	65.8 – 85.4	<0.0001
% Change in RM Score	48	42.0	56.1	24.5 – 59.6	<0.0001	41	41.5	53.8	21.1 – 61.9	0.0002
% Change in Current Pain	45	48.8	83.3	25.7 – 72.0	0.0001	40	38.4	100.0	0.1 – 76.8	0.0497
% Change in Average Pain	43	27.2	33.3	6.7 – 47.8	0.0106	41	51.7	75.0	33.5 – 69.9	<0.0001
% Change in Worst Pain	47	34.8	20.0	22.3 – 47.3	<0.0001	45	44.7	57.9	28.7 – 60.7	<0.0001

Legend: RM – Roland Morris Low Back Pain and Disability questionnaire; FU – follow up; CI – confidence interval for mean

## References

[B1] Arbit EPS (2001). Lumbar stenosis: A clinical review. Clin Orthop.

[B2] LaBan MMIA (2003). "Young" lumbar spinal stenotic: review of 268 patients younger than 51 years.. Am J Phys Med Rehabil.

[B3] Jenis LG, An HS (2000). Lumbar foraminal stenosis. Spine.

[B4] Matsumoto T, Yoshida M, Yamada H, Kurimoto K, Tamaki T (2001). Lumbar canal stenosis caused by hypertrophy of the posterior longitudinal ligament case report. Spine.

[B5] Adamova B, Vohanka S, Dusek L (2003). Differential diagnostics in patients with mild lumbar spinal stenosis the contributions and limits of various tests. Eur Spine J.

[B6] Szpalski MGR (2003). Lumbar spinal stenosis in the elderly: an overview. Eur Spine J.

[B7] Gibson JNAWGGIC (2004). Surgery for degenerative lumbar spondylosis. The Cochrane Library.

[B8] Binder DKSMHWPR (2002). Lumbar spinal stenosis. Sem Neurol.

[B9] Özaktay ACCJMAIDLJAWJN (2002). Dorsal root sensitivity to interleukin-1 beta, interleukin-6 and tumor necrosis factor in rats. Eur Spine J.

[B10] Anzai H, Hamba M, Onda A, Kikuchi S, Konno S (2002). Epidural application of nucleus pulposus enhances nociresponses of rat dorsal horn neurons. Spine.

[B11] Lipetz JS (2002). Pathophysiology of inflammatory, degenerative and compressive radiculopathies. Phy Med Rehabil Clin N Am.

[B12] Hoyland JA, Freemont AJ, Jayson MIV (1989). Intervertebral foramen venous obstruction: a cause of periradicular fibrosis. Spine.

[B13] Porter RW (1996). Spinal stenosis and neurogenic claudication. Spine.

[B14] Kobayashi S, Shizu N, Suzuki Y, Asai T, Yoshizawa H (2003). Changes in nerve root motion and intradicular blood flow during and intraoperative straight-leg-raising test. Spine.

[B15] Schonstrom NLSWJHT (1989). Dynamic changes in the dimensions of the lumbar spinal canal: an experimental study in vitro. J Orthop Res.

[B16] Beurskens AJ, de Vet HC, Koke AJ, van der Heijden GJ, Knipschild PG (1995). Measuring the functional status of patients with low back pain assessment of the quality of four disease-specific questionnaires. Spine.

[B17] Deyo RA (1988). Measuring the functional status of patients with low back pain. Arch Phys Med Rehabil.

[B18] Cox JM (1999). Low back pain: mechanisms, diagnosis and treatment.

[B19] Stern PJ, Cote P, Cassidy JD (1995). A series of consecutive cases of low back pain with radiating leg pain treated by chiropractors. J Manipulative Physiol Ther.

[B20] Gudavalli MR, Cox JM, Cramer GD, Baker JA, Patwardhan AG (1997). Intervertebral disc pressure changes during a chiropractic procedure. BED- Advances in Bioengineering.

[B21] Gudavalli MR, Cox JM (1999). Biomechanics research on flexion-distraction procedure. Low Back Pain: Mechanism, Diagnosis and Treatment.

[B22] Hall TM, Elvey RL (1999). Nerve trunk pain physical diagnosis and treatment. Man Ther.

[B23] Butler DS (2000). The Sensitive Nervous System.

[B24] Lewit K, Liebenson C (1993). Palpation - problems and implications. J Manipulative Physiol Ther.

[B25] McGill S (2002). Low Back Disorders.  Evidence-Based Prevention and Rehabilitation.

[B26] Richardson CJGHPHJ (1999). Therapeutic Exercise For Spinal Segmental Stabilization In Low Back Pain.  Scientific Basis and Clinical Approach..

[B27] McGill SM (2001). Low back stability: from formal description to issues for performance and rehabilitation. Exerc Sport Sci Rev.

[B28] Hurwitz ELMHHPKGFBTRYFAAH (2002). A randomized trial of medical care with and without physical therapy and chiropractic care with and without physical modalities for patients with low-back pain: Six-month follow-up outcomes from the UCLA Low-Back Pain Study. Spine.

[B29] Hashemi LWBSCEA (1998). Trends in disability duration and cost of workers’ compensation low back pain claims (1988-1996). J Occup Environ Med.

[B30] Hsieh CJPRBAAHPMH (1992). Functional outcomes of low back pain: Comparison of four treatment groups in a randomized controlled trial. J Manipulative Physiol Ther.

[B31] Salaffi FSASCACGW (2004). Minimal clinically important changes in chronic musculoskeletal pain intensity measured on a numerical rating scale. Eur J Pain.

[B32] Johnsson KE, Rosen I, Uden I (1992). The natural course of lumbar spinal stenosis. Clin Orthop.

[B33] Atlas SJDRAKRBCAMPDLLJMSDE (1996). The Maine Lumbar Spine Study. Part III. 1-year outcomes of surgical and nonsurgical management of lumbar spinal stenosis. Spine.

[B34] Atlas SJ, Keller RB, Robso ND, Deyo RA, Singer DE (2000). Surgical and nonsurgical management  of lumbar spinal stenosis four-year outcomes from the Maine Lumbar spine study. Spine.

[B35] Simotas AC, Dorey FJ, Hansrai KK, Cammisa F (2000). Nonoperative treatment for lumbar spinal stenosis clinical and outcome results and a 3-year survivorship analysis. Spine.

[B36] Amundsen TWHNHJMBAMLF (2000). Lumbar spinal stenosis: Conservative or surgical managment?. Spine.

[B37] Pickar JG, McLain RF (1995). Responses of mechanosensitive afferents to manipulation of the lumbar facet in the cat. Spine.

[B38] Leinonen V, Maatta S, Taimela S, Herno A, Kankaanpaa M, Partanen J, Kansanen M, Hanninen O, Airaksinen O (2002). Impaired lumbar movement perception in association with postural stability and motor and somatosensory-evoked potentials in lumbar spinal stenosis. Spine.

[B39] Sanders GEROTRMP (1990). Chiropractic adjustive manipulation on subjects with acute low back pain: visual analog pain scores and plasma b-endorphin levels. J Manipulative Physiol Ther.

[B40] Bulbulian R, Burke J, Dishman JD (2002). Spinal reflex excitability changes after lumbar spine passive flexion mobilization. J Manipulative Physiol Ther.

[B41] Leboeuf-Yde C, Hennius B, Rudberg E, Leufvenmark PTM (1997). Side effects of chiropractic treatment a prospective study. J Manipulative Physiol Ther.

[B42] Senstad O, Leboeuf-Yde C, Borchgrevink C (1997). Frequency and characteristics of side effects of spinal manipulative therapy. Spine.

